# Chorioamnionitis following preterm premature rupture of membranes and fetal heart rate variability

**DOI:** 10.1371/journal.pone.0184924

**Published:** 2017-09-25

**Authors:** Laurent Vandenbroucke, Matthieu Doyen, Maëla Le Lous, Alain Beuchée, Philippe Loget, Guy Carrault, Patrick Pladys

**Affiliations:** 1 INSERM, UMR1099, Signal and Image Processing Laboratory, SEPIA team, Rennes, France; 2 CHU Rennes, Department of Obstetrics, University Hospital of Rennes, Rennes, France; 3 INSERM, U1414, Clinical Investigation Center, Rennes, France; 4 Univ Rennes 1, Faculté de Médecine, Rennes, France; 5 CHU Rennes, Department of Pediatrics, University Hospital of Rennes, Rennes, France; 6 CHU Rennes, Department of Anatomical Pathology, University Hospital of Rennes, Rennes, France; University of British Columbia, CANADA

## Abstract

**Introduction:**

The objective of this study was to identify prenatal markers of histological chorioamnionitis (HC) during pPROM using fetal computerized cardiotocography (cCTG).

**Materials and methods:**

Retrospective review of medical records from pregnant women referred for pPROM between 26 and 34 weeks, in whom placental histology was available, in a tertiary level obstetric service over a 5-year period. Fetal heart rate variability was assessed using cCTG. Patients were included if they were monitored at least six times in the 72 hours preceding delivery. Clinical and biological cCTG parameters during the pPROM latency period were compared between cases with or without HC.

**Results:**

In total, 222 pPROM cases were observed, but cCTG data was available in only 23 of these cases (10 with and 13 without HC) after exclusion of co-morbidities which may potentially perturb fetal heart rate variability measures. Groups were comparable for maternal age, parity, gestational age at pPROM, pPROM duration and neonatal characteristics (p>0.1). Baseline fetal heart rate was higher in the HC group [median 147.3 bpm IQR (144.2–149.2) vs. 141.3 bpm (137.1–145.4) in no HC group; p = 0.02]. The number of low variation episodes [6.4, (3.5–15.3) vs. 2.3 (1–5.2); p = 0.04] was also higher in the HC group, whereas short term variations were lower in the HC group [7.1 ms (6–7.4) vs. 8.1 ms (7.4–9); p = 0.01] within 72 hours before delivery. Differences were especially discriminant within 24 hours before delivery, with less short-term variation [5 ms (3.7–5.9) vs. 7.8 ms (5.4–8.7); p = 0.007] and high variation episodes [3.9 (4.9–3.2) vs. 0.8 (1.5–0.2); p < 0.001] in the HC group.

**Conclusion:**

These results show differences in fetal heart rate variability, suggesting that cCTG could be used clinically to diagnoses chorioamnionitis during the pPROM latency period.

## Introduction

Preterm premature rupture of membranes (pPROM) occurs in 2 to 3% of pregnancies and is associated with a high frequency of preterm deliveries and neonatal morbidity and mortality [1]. One of the main risks associated with pPROM is ascending infection of the amniotic cavity. Histological chorioamnionitis (HC), leading to fetal inflammatory response syndrome, is associated with neonatal morbidity (including a high rate of cerebral palsy, intracranial hemorrhage, sepsis, pneumonia, necrotizing enterocolitis, and death), even in infants born at term [2–4]. Studies in recent years have failed to identify a satisfactory prenatal marker of infection for the early diagnosis of chorioamnionitis. Guidelines for the management of women with pPROM do not thus take prenatal markers of infection into account and are mainly based on the gestational age at which pPROM occurs [5]. It is therefore important to identify non-invasive, easily accessible markers of high sensitivity and specificity for the early diagnosis of chorioamnionitis during the pPROM latency period. Many studies have reported early changes in heart rate variability in cases of inflammation or during infection, especially in neonates and premature newborns [6–9]. We postulated that histological chorioamnionitis could lead to significant differences in fetal heart rate variability. We retrospectively investigated fetal computerized cardiotocography (cCTG) parameters during the last 72 hours of the pPROM latency period to quantify changes in fetal heart rate variability as a proof of concept.

## Materials and methods

Ethics approval for this study was obtained from the local research ethics committee of Rennes (14.76, 10 November 2014). In this retrospective study, we analyzed the files of women with a singleton pregnancy who were referred for pPROM to the Obstetrics Department of Rennes University Hospital (level III maternity unit, 4000 births per year) between November 2007 and November 2012. The inclusion criteria were: occurrence of pPROM between 26 and 34 WG (weeks of gestation), gestational age confirmed by measuring crown–rump length on first-trimester ultrasound, latency period between pPROM and delivery ≥ 72 hours and available sample of the placenta for histological analysis, six consecutive cCTG recordings made before spontaneous or induced delivery, last cCTG performed within the last 24 hours prior to delivery. The exclusion criteria were: multiple pregnancy, evidence of placental anomalies or major structural fetal anomalies, preexisting or gestational diabetes mellitus, active smoking, intrauterine growth restriction, induction of labor and gestational age at delivery > 34 WG. These exclusion criteria were chosen to obtain a subgroup of isolated pPROM without comorbidities and to avoid most of the factors that interact with fetal heart rate variability [10,11]. All women received antibiotic prophylaxis (intravenous amoxicillin or erythromycin) and corticosteroid therapy (two doses of intravenous betamethasone 12 mg) at admission following evidence-based guidelines for pPROM. During the study period, pPROM was diagnosed by physical examination with a sterile speculum, following obvious leakage of amniotic fluid from the cervical os, and confirmed by an IGFBP-1 test. A fetal cardiotocography (CTG) was performed twice daily (morning and evening) for 30 min to assess fetal well-being and C-reactive protein measurements were performed three times a week. The midwives used standard (Philips Avalon FM20®) or computerized (Sonicaid Oxford 8002® system—Ltd, Chichester, UK) CTG for fetal monitoring during the study period. If chorioamnionitis was clinically suspected (non-reassuring fetal heart rate, uterine contraction, maternal fever, stained amniotic fluid, C-reactive protein > 20 mg/L, hyperleukocytosis > 15,000/mm^3^), an elective cesarean section was performed. In all other cases, delivery was spontaneous.

The diagnosis of HC was performed by an experienced anatomical pathology physician and was defined as acute inflammation (neutrophil infiltration) of the membranes and chorion on microscopic examination [12]. An increase of neonatal C-reactive protein levels combined with clinical signs of infection and positive blood culture with pathogenic bacteria defined early-onset neonatal sepsis.

The cCTG parameters were collected every 12 h at the same time each day by a specific monitor with a sample frequency of 4 Hertz (Sonicaid Oxford 8002® system—Ltd, Chichester, UK). The parameters measured were those initially described by G.S. Dawes and C.W. Redman [13]: baseline heart rate in beats per minute (bpm), number of accelerations of 10 and 15 bpm for at least 15 s, number of decelerations exceeding 20 bpm for at least 30 s, duration of episodes of high and low variation in minutes, short-term variation (STV) in milliseconds, and number of fetal movements per hour. Briefly, the method used to calculate STV was as follows. The recording was divided into one-minute intervals. Intervals containing a deceleration or part of a deceleration were discarded, as were intervals with high signal loss or artefacts. Each remaining interval was divided into sixteen epochs of 3.75 seconds. The mean fetal heart rate for each epoch was determined and expressed as a pulse interval in milliseconds. The difference between adjacent epochs was calculated. The STV was calculated as the mean of adjacent epoch pulse intervals over the recording during all valid minutes. The episodes of high and low variation were defined as sections of the trace in which the one-minute peak-to-peak variation was respectively above 32 ms or below 30 ms for 5 of 6 consecutive minutes.

We used a *post-hoc* temporal index in which the mean value of the two last recordings was divided by the mean value of all the preceding recordings to discriminate changes in STV and high variation episodes occurring during the very last recordings before delivery and those occurring during other recordings performed during the pPROM latency period.

Results are expressed as the median and interquartile range (IQR). The Mann-Whitney U test and Fisher’s exact test were used, as appropriate, to compare clinical and biological characteristics and cCTG parameters between pPROM with HC and pPROM without HC (control) groups. cCTG parameters were compared using the mean of the whole recordings, the last recording before delivery, and the *post hoc* temporal index. The diagnostic value was estimated using area under the Receiver Operating Characteristic (ROC) curves. A *p* value < 0.05 was considered to be statistically significant. All analyses were performed using *R* (www.r-project.org) and Matlab® (version R2012b 8.0.0.783, The MathWorks Inc.).

## Results

**Fig 1:**
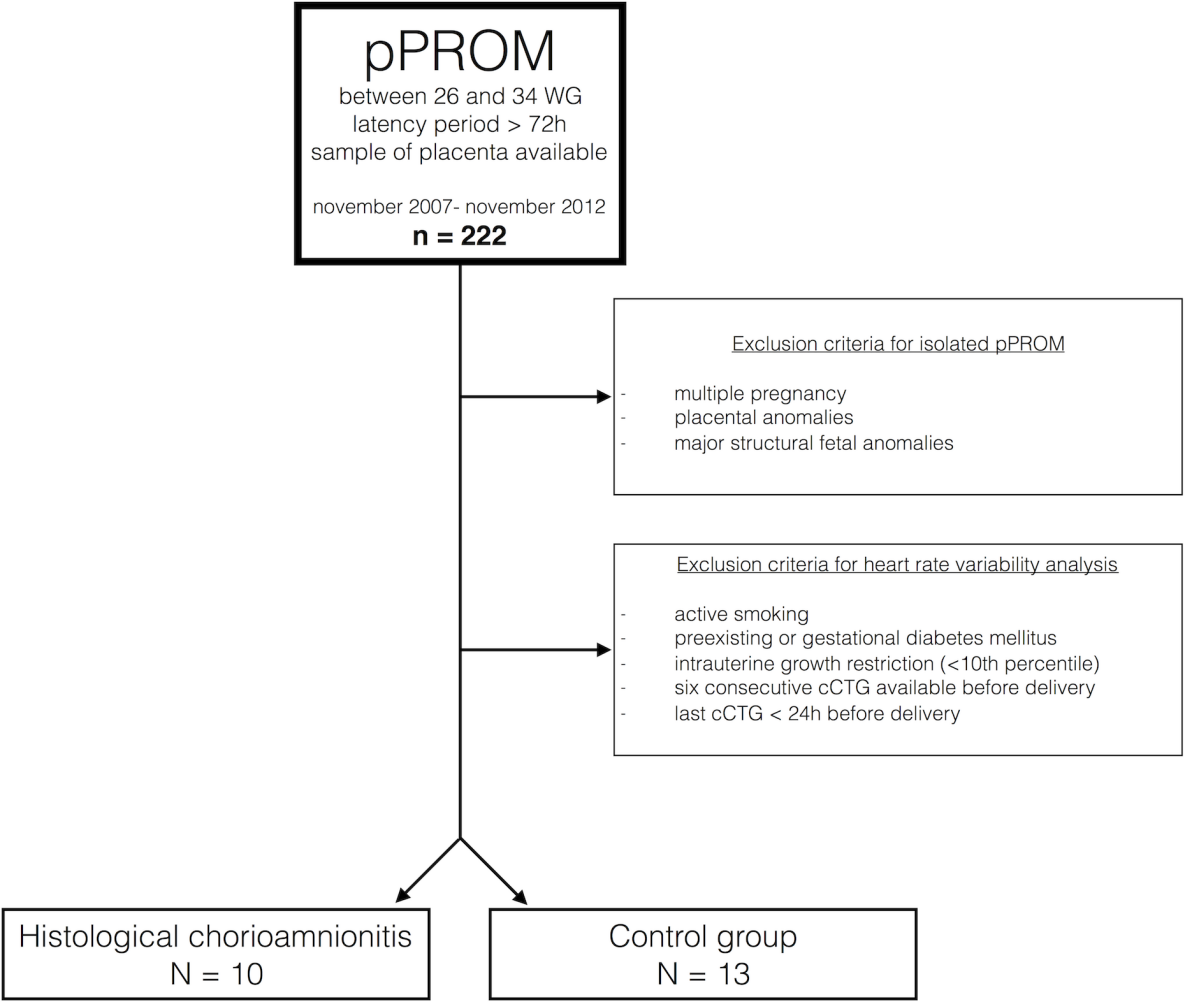
Flowchart of the study.

During the study period, 23 of the 222 women admitted for pPROM between 26 to 34 WG fulfilled the inclusion and exclusion criteria (Fig 1).

**Fig 2:**
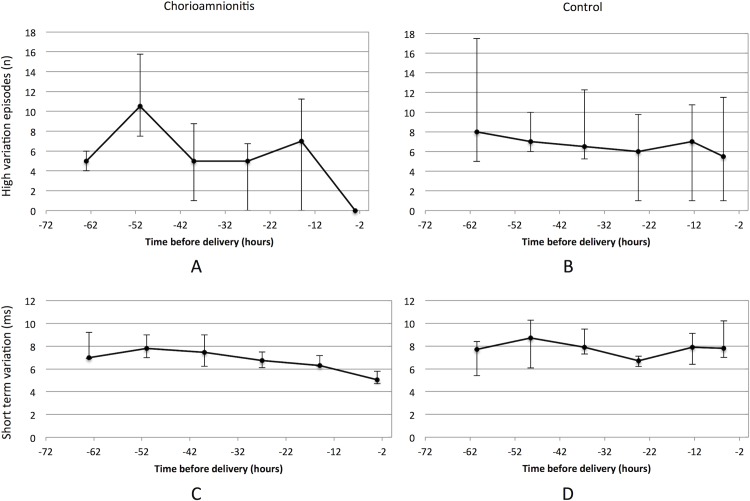
Linear representation of high variation episodes (figures A and B; median and interquartile range) and STV (figures C and D; median and interquartile range) during the six last cCTG before delivery. The cCTG parameters were collected every 12 h at the same time (around 8:00 a.m. and p.m.).

The clinical and biological variables of the two groups did not significantly differ, despite a tendency towards a lower gestational age at pPROM and delivery and higher neonatal morbidity in the HC group (Table 1). Baseline heart rate and the number of low variation episodes were higher in cases of HC than controls (Table 2). STV was lower in cases of HC than controls, especially for recordings made within 24 hours before delivery (Fig 2).

**Table 1:** Clinical characteristics in cases of pPROM with or without HC.

**Maternal characteristics**	**Chorioamnionitis** **(n = 10)**	**Control** **(n = 13)**	**p**
Maternal age (years)	31 (26.5; 35)	32 (28; 36)	0.64
Gestational age at pPROM (WG)	29.7 (28; 31)	31.3 (27.9; 31)	0.42
Parity	1.5 (1; 2.3)	1 (1; 2.3)	0.76
Interval PROM-delivery (days)	8.5 (3; 10.5)	6 (3.5; 10.5)	0.95
Clinical suspicion of chorioamnionitis	80%	46%	**0.01**
Lag time last recording/delivery (hours)	3 (2; 8)	5.5 (4; 8)	0.24
Lag time last corticosteroid injection/delivery (days)	6 (2.8; 10)	4 (2.8; 10)	0.26
**Neonatal characteristics**			
Gestational age at birth (WG)	31.4 (28.6; 32.6)	32.1 (30; 32.6)	0.29
Birth weight (g)	1600 (1037.5; 1900)	1735 (1320; 2247.5)	0.26
Umbilical pH at birth	7.30 (7.24; 7.35)	7.29 (7.25; 7.35)	0.82
Apgar score at 1 minute	7 (5; 9.8)	9 (7.3; 10)	0.68
Apgar score at 5 minutes	9 (7.3; 10)	10 (8.5; 10)	0.86
Neonatal death	10% (n = 1)	0%	0.43
Duration of assisted ventilation (days)	0.88 (0; 1)	1.25 (0; 1.5)	0.61
Duration of hospitalization (days)	45.5 (34.7; 57)	33 (19; 45.7)	0.23
Proven early-onset sepsis	20% (n = 2)	0%	0.18

WG: weeks of gestation; pPROM: preterm premature rupture of membranes

Results are expressed as the median and interquartile range (25%; 75%). The Mann-Whitney U test and Fisher’s exact test were used, where appropriate.

**Table 2:** Biological and cCTG parameters in cases of pPROM with or without HC.

**Biological parameters**	**Chorioamnionitis (n = 10)**	**Control** **(n = 13)**	**p**
C-Reactive protein at admission (mg/l)	2 (2; 5.8)	4 (3; 16)	0.22
Leucocytes count at admission (G/mm^3^)	11.4 (10; 12.4)	12 (10.4; 13)	0.77
Last C-Reactive protein before delivery	8 (5; 17.3)	4 (1.6; 15)	0.2
Bacterially positive vaginal swab	30%	33.3%	1
**cCTG parameters**			
**Recordings of the last 72h before delivery**			
No. of fetal movements/h	29.9 (16.8; 48.1)	23.3 (8.6; 63.8)	0.78
Baseline heart rate (bpm)	147.3 (144.2; 149.2)	141.3 (137.1; 145.4)	**0.02**
No. of accelerations > 10 bpm/15s	3.7 (2.7; 5)	4 (2.6; 5)	0.76
No. of accelerations > 15 bpm/15s	0.8 (0.3; 2)	1 (0.6; 3.3)	0.32
No. of decelerations > 20 bpm/30s	0.2 (0; 0.3)	0.2 (0; 0.2)	0.82
Episodes of high variation (n)	6.3 (3.8; 7.4)	9.2 (6.2; 11.6)	0.06
Episodes of low variation (n)	6.4 (3.5; 15.3)	2.3 (1; 5.2)	**0.04**
Short term variation (ms)	7.1 (6; 7.4)	8.1 (7.4; 9)	**0.01**
**Last recording before delivery**			
Short term variation of the last recording (ms)	5.1 (3.7; 5.9)	7.8 (5.4; 8.7)	**0.007**
Temporal indices*			
Short term variation temporal index (ms)	0.7 (0.7; 0.9)	1.1 (0.9; 1.2)	**0.003**
High variation temporal index	-3.9 (-4.9; -3.2)	-0.8 (-1.5; -0.2)	**< 0.001**

WG: weeks of gestation; pPROM: preterm premature rupture of membranes; cFHR: computerized fetal heart rate; bpm: beats per minute; s: second; ms: milliseconds

* Mean value of the last two recordings divided by the mean value of the preceding recordings

Results are expressed as the median and interquartile range (25%; 75%). The Mann-Whitney U test and Fisher’s exact test were used, where appropriate.

HC was also associated with a low temporal index both for STV and high variation episodes. The diagnostic value of the temporal index for high variation episodes for HC in cases of pPROM was as follows: sensitivity 90%, specificity 84.6%, positive predictive value 71.5%, negative predictive value 95.2%, AUC = 0.88 (IC 95% 0.73 to 100).

## Discussion

In this retrospective study on prolonged pPROM (i.e. > 72 h) occurring between 26 and 34 WG, we have confirmed the low diagnostic value of the prenatal markers usually used in clinical practice for the early diagnosis of chorioamnionitis. We identified significant differences in cCTG parameters in cases of histologically confirmed chorioamnionitis versus controls. We observed a decrease number of STV and high variation episodes (recorded 24 to 48 hours before delivery) in association with chorioamnionitis.

Our study was limited to the patients with a pPROM latency period of at least three days before delivery, because we wanted to observe the changes in prenatal markers during this period. As expected, this limited the sample size, in agreement with the reported frequency of prolonged pPROM (42% in the study of Pasquier J.C. et al) [14]. Moreover, we did not include patients with diabetes mellitus or those who were persistent active smokers (as these factors are known to influence fetal heart rate variability [10,11]) to allow identification of significant changes in heart rate variability specifically associated with HC.

We used cCTG parameters from the Sonicaid Oxford 8002® system (Ltd, Chichester, UK) to retrospectively assess fetal heart rate variability, without access to beat-to-beat recordings. We have therefore not studied the other methods used to quantify heart rate variability. Using only cCTG parameters limits the assessment of fetal heart rate variability to that in the time domain and does not allow spectral or nonlinear analyses. Moreover, the low sample frequency does not allow the sensitive evaluation of STV that can be obtained through post-natal ECG recording. Despite these limitations, we observed very significant changes in fetal heart rate variability associated with HC. This confirmed our initial hypothesis that significant changes in fetal heart rate variability occur in cases of HC during the pPROM latency period. Our results are consistent with those of previous studies performed on premature newborns or in animal models with extensive heart rate variability analysis. Indeed, the fetal inflammatory response contributes directly (via effects on sinoatrial node pacemaker cells) or indirectly (via autonomic nervous system activation and dysfunction) to time-dependent changes in heart rate variability, even before the occurrence of clinical signs [15–21].

This is the first study that attempts to evaluate fetal heart rate variability to assess the risk of HC during the pPROM latency period. Buscicchio et al. examined cCTG at admission in cases of pPROM occurring between 34 and 36 WG, and compared them (n = 100) to healthy controls matched for age, parity, and gestational age [22,23]. They found that STV was lower in pPROM and that both the number of low variation episodes and baseline heart rate were higher in cases of pPROM than in controls. These results are consistent with our findings, although the inclusion criteria and methods were different. Their study was limited to cCTG at admission for pPROM. Furthermore, they did not histologically evaluate chorioamnionitis, and simply compared cases of pPROM to healthy controls. Buhimschi et al. visually analyzed CTG recordings (following NICHD guidelines) obtained at admission, at amniocentesis (performed before planned or emergency delivery to rule-out infection/inflammation), and before delivery, of pregnant women with preterm labor and pPROM [24]. They observed that the baseline heart rate was higher in cases of severe intra-amniotic inflammation than controls throughout the entire monitoring period, and that a non-reassuring CTG on admission was a specific, but not a sensitive, predictor of early-onset neonatal sepsis (EONS).

The results concerning biological parameters of this study are consistent with those of recent studies that failed to identify a satisfactory prenatal marker of infection for the prediction of chorioamnionitis (reviewed in 4). This suggest that chorioamnionitis is often only apparent at an advanced stage of infection. Indeed, our results confirm that biological tests (i.e. C-reactive protein and leukocytosis) have poor diagnostic value [25]. Inflammatory cytokines, such as IL-6 in the maternal blood, amniotic fluid, or vaginal discharge, may be promising markers, but their sampling is invasive and they are still under evaluation.

## Conclusion

We found that several parameters of cCTG significantly differ during the last days before delivery between women with or without HC, even though cCTG only provides a simple analysis of fetal heart rate variability. The main changes observed were decreases in the n umber of high variation episodes (*p* < 0.001) and STV (*p* = 0.003). These data suggest, for the first time, that fetal heart rate variability could provide an early evaluation of the risk of HC during the pPROM latency period. This is consistent with the diagnostic value of heart rate variability measurements observed in studies concerning late onset neonatal sepsis, necrotizing enterocolitis, or in an animal model of inflammatory response syndrome [11–17]. It suggests that fetal heart rate variability, which is a non-invasive and easily accessible tool, could be a useful prenatal marker for the early diagnosis of chorioamnionitis during pPROM, but this needs to be prospectively confirmed.
